# Clinical features, treatment outcomes and mortality risk of tuberculosis sepsis in HIV-negative patients: a systematic review and meta-analysis of case reports

**DOI:** 10.1007/s15010-022-01950-4

**Published:** 2022-11-16

**Authors:** Bayode R. Adegbite, Nadege O. M. Elegbede-Adegbite, Jean R. Edoa, Yabo J. Honkpehedji, Jeannot F. Zinsou, Jean Claude Dejon-Agobé, Ayola A. Adegnika, Martin P. Grobusch

**Affiliations:** 1 Centre de Recherches Médicales de Lambaréné and African Partner Institution, Lambaréné, Gabon; 2 Department of Infectious Diseases, Center of Tropical Medicine and Travel Medicine, Amsterdam University Medical Centers, Location AMC, Amsterdam Public Health, Amsterdam Infection & Immunity, University of Amsterdam, Amsterdam, The Netherlands; 3 Institut für Tropenmedizin, Universität Tübingen and German Center for Infection Research, Tübingen, Germany; 4 Centre de Dépistage et de Traitement de l’Ulcère de Buruli de Lalo, Ministére de la Santé du Bénin, Lalo, Benin; 5 Department of Parasitology, Leiden University Medical Center, Leiden, The Netherlands; 6 Masanga Medical Research Unit (MMRU), Tonkolili, Sierra Leone; 7 Institute of Infectious Diseases and Molecular Medicine (IDM), University of Cape Town, Cape Town, South Africa

**Keywords:** Sepsis, Tuberculosis septic shock, Tuberculosis in intensive care unit, Case fatality for tuberculosis septic shock, Tuberculosis sepsis

## Abstract

**Purpose:**

Tuberculosis sepsis (TBS) is sepsis due to the *Mycobacterium* species causing tuberculosis (TB). It seems to be rare in HIV-negative patients and mainly individual case reports have been reported. This systematic review summarizes the epidemiology, clinical features, and treatment outcomes of TBS in HIV-negative patients.

**Methods:**

An electronic search of PubMed, Embase, Web of Science, and Google Scholar was performed to identify published case reports of TBS between January 1991 and September 2022.

**Results:**

Twenty-five articles reported 28 cases of TBS in HIV-negative patients, among which 54% (15/28) were women; with 50% (14/28) of patients not having reported predisposing factors. A total of 64% (18/28) of patients died, and the diagnosis was obtained for many of them only post-mortem. Two of the reports mentioned the BCG vaccination status. A higher proportion of deaths occurred in patients with delayed diagnosis of sepsis. The probability of survival of patients diagnosed with tuberculosis sepsis was 68% on day 10; 41% on day 20; and 33% on day 30 after admission.

**Conclusions:**

Our review showed TBS occurred in HIV-negative patients and some of them have no known immunocompromised underlying co-morbidity. TBS might not be rare as clinicians thought but might be prone to be missed. In endemic settings, *M. tuberculosis* etiology of sepsis should be accounted for early, irrespective of HIV infection status.

**Supplementary Information:**

The online version contains supplementary material available at 10.1007/s15010-022-01950-4.

## Introduction

Sepsis and tuberculosis kill around 11 million [1] and 1.5 million people per year [2], respectively. Tuberculosis sepsis (TBS), also known as sepsis tuberculosis gravissima, was first described by Landouzy in 1908 [3]. TBS has been mostly reported in HIV-infected patients [4–7]; however, it can also occur in immunocompetent patients [8, 9].

It has been estimated that half of TBS remains undiagnosed at the time of death [10]. An analysis of the United States’ databases of patients with sepsis showed that fifteen percent of patients did not have clinical signs and symptoms leading to suspect sepsis on admission [11]. The mortality rate was worst in this group of patients as compared to the group of patients with sepsis at presentation [11]. Prompt diagnosis and early treatment are key to the management of sepsis. Delay in antibiotics administration is associated with the worsening of sepsis severity both in sepsis in general and in TB sepsis in particular [12, 13].

TBS carries a fatal prognosis because it is overlooked by clinicians; mainly in the case of patients without HIV infection [10]. A case series reported by Kethireddy et al. suggests that most TBS patients died [13], likely because TBS is not coming into the mind of the clinician as an alternative diagnostic or etiology of organ failure. These findings highlight the critical need to improve clinicians’ awareness of TBS. This systematic review and meta-analysis aim to better understand the epidemiology, clinical features, and factors associated with the treatment outcome of TBS in HIV-negative patients.

## Methods

### The search strategy and inclusion criteria

The review was undertaken and reported by following the preferred reporting items for systematic review and meta-analysis (PRISMA 2020 and PRISMA-S guidelines [14, 15]. The protocol of the review was registered with PROSPERO (CRD42022296768).

An electronic search of the published literature was conducted on December 1, 2021, and updated on September 25, 2022, in PubMed, Embase, Web of Science (core collection) and Google Scholar to identify case reports or case series of tuberculosis sepsis. As suggested by PRISMA-S [15] and Bramer and collaborators [16], the first 200 results on Google Scholar were selected. We also searched the reference lists of the included case reports. The following search terms were used in PubMed:

("tuberculosis"[All Fields] OR "tuberculosis"[MeSH Terms] OR "tuberculosis"[All Fields] OR "tuberculoses"[All Fields] OR "tuberculosis s"[All Fields]) AND ("sepsis"[MeSH Terms] OR "sepsis"[All Fields]) AND ((("ieee int conf automation sci eng case"[Journal] OR "case phila"[Journal] OR "case"[All Fields]) AND "report*"[All Fields]) OR (("ieee int conf automation sci eng case"[Journal] OR "case phila"[Journal] OR "case"[All Fields]) AND "serie*"[All Fields])). The full description of the search strategy of the others databases used is reported in Supplementary File S1. Additionally, we conducted a cross-reference analysis to retrieve manuscripts that were not identified during our initial search. With the purpose of uniformly applying consensus criteria for the definition of sepsis, we restricted the case reports or series to be included to those published after the first consensus definition of sepsis by the American College of Chest Physicians and the Society of Critical Care Medicine (1991) [17, 18]. We excluded case reports (a) with unclear clinic-pathological data of the diagnosis of sepsis or lack of information on the diagnostic method and treatment outcome; (b) duplicate cases using Rayyan platform [19]; (c) TBS cases in HIV-infected patients; (d); case reports in languages other than English or French; (e) tuberculosis bloodstream infections not fulfilling sepsis criteria; (f) sepsis cases due to *Mycobacterium* species other than *Mycobacterium tuberculosis*, *M. bovis* and *M. africanum*; and (g) new-borns or infants with congenital tuberculosis not reported as tuberculosis sepsis. The titles and abstracts were initially screened independently by two reviewers (EJR and NOE). The full texts of the relevant articles were assessed for inclusion by two independent reviewers (BRA and NOE) using the Rayyan platform [19]. The agreement of both reviewers was required for inclusion and exclusion. Any disagreement was resolved by consensus. If BRA and NOE did not agree after discussion, a third investigator (YJH) was consulted. The full list of excluded cases is reported in Supplementary File S2.

### Data extraction and quality assessment

The following data were extracted from the original studies: first author; year of publication; country of origin; study population and participant demographics and baseline characteristics; clinical features, outcomes, and times of measurement. BRA and NOE independently extracted data using the items pre-defined on the excel sheet. The quality of included studies was assessed with the Joanna Briggs Institute Critical Appraisal Checklist for Case Reports [20], which consists of eight yes/no/unclear questions which led to the overall appraisal: ‘Include’, ‘Exclude’ or ‘Seek further info’.

### Summary measures and statistical analysis

Descriptive statistics of publication characteristics and patient demographic variables were performed. Case report data were grouped by type of patients (adult or infant). The patients’ sociodemographic data were presented separately for adults and infants. The means of age, time to diagnosis of tuberculosis sepsis, time to initiation of an empiric anti-tuberculosis treatment and corresponding standard deviation (SD) were described for each category of patients. To better describe factors associated with death; the clinical features, the predisposing co-morbidities, and initial diagnosis were ranged into three categories maximum. Fisher’s exact test was used to identify factors significantly associated with death. Cox’s regression logistic was not performed as planned in the protocol due to the small number of cases.

## Results

**Fig. 1:**
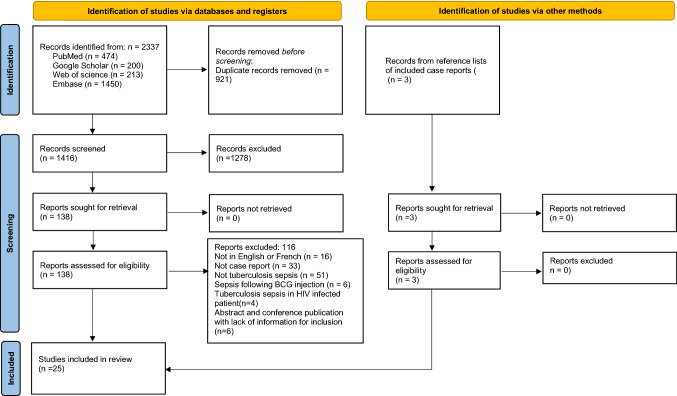
Flowchart of the study selection process

The initial database search identified 2337 articles, of which 1416 were screened using the abstract and title. A total of 137 full articles were assessed for eligibility and 25 articles (28 individual case reports) were included in the final analysis (Fig. 1). All case reports included in this review met the ‘Include’ overall appraisal using the Joanna Briggs Institute tool and were classified as Low risk of bias (see Supplementary File S3).

### Patient demographics, co-morbidities, predisposing factors, and clinical manifestation

Among the total of 28 individual cases [8, 9, 21–43], four of them were infants (Table1). The mean age of the infants was 23 days (SD = 9). The mean age of adults was 44 years (SD = 18). A total of 15/28 (54%) cases were women. For 50% (14/28) of the patients, no pre-disposing factors were reported. BCG vaccination status was only reported in two cases. The main chief complaints were ‘weakness’ and ‘dyspnoea’, Table 2. A total of 61% (17/28) of patients reported cough, and all of the patients had a fever, respiratory distress, and hypotension. Leukopenia was the most common laboratory abnormality reported.

**Table 1:** Overview of included studies and descriptive data

Authors	Years	Title	Sex	Category	BCG Vaccination	Age (Days for Infant/ Year for adult)	First diagnosis	Comorbidity	Treatment outcome	Site of tuberculosis infection	Bacteriological investigation
Artsiom et al.	2020	A Case of Miliary Perinatal Tuberculosis in a Preterm Newborn Infant Presenting as Peritonitis	M	Infant	Yes	25	Peritonitis	No	Discharged	Pulmonary Tuberculosis	Gastric aspirate culture: Positive
Nakbanpot et al.	2013	Congenital Tuberculosis because of Misdiagnosed Maternal Pulmonary Tuberculosis during Pregnancy	F	Infant	Not reported	19	Sepsis	Prematurity	Death	Pulmonary Tuberculosis	Sputum culture: Positive
Barbosa et al.	2013	Disseminated hematogenous tuberculosis in puerperium—case report	F	Adult	Not reported	22	Chorioamnionitis	Pregnancy	Death	Pulmonary and extra pulmonary Tuberculosis	Post-mortem pathology of lung, liver, uterus: giant cells, caseous necrosis and acid-fast bacilli
Chun-Yuan et al.	2016	Disseminated tuberculosis presenting as tuberculous peritonitis and sepsis tuberculosa gravissima in a patient with cirrhosis of the liver: A diagnosis of challenge	M	Adult	Not reported	81	Peritonitis	Cirrhosis	Death	Pulmonary and extra pulmonary Tuberculosis	Blood, ascites, and sputum culture: Positive
Kindler et al.	2001	Fatal sepsis due to *Mycobacterium tuberculosis* after allogeneic bone marrow transplantation	M	Adult	Not reported	34	CMV reactivation	Bone marrow transplantation	Death	Pulmonary Tuberculosis and extra pulmonary TB	Tracheal aspirates culture: Positive
Mitchon et al.	2017	Fatal Sepsis from *Mycobacterium tuberculosis* In An HIV-Negative Alcoholic Female	F	Adult	Not reported	28	Sepsis	No	Death	Pulmonary and extra pulmonary Tuberculosis	Excisional biopsy of a chest wall lymph node: necrotising granulomas Ascites punction culture: Positive
Sydow et al.	1992	Multiple organ failure in generalized disseminated tuberculosis	M	Adult	Not reported	72	Sepsis	No	Death	Pulmonary and extra pulmonary Tuberculosis	Post-mortem cultures of multi-organ (lungs, liver, adrenal cortex, both kidneys, spleen): Positive
Okascharoenet al.	2003	Neonatal Tuberculosis Associated With Shock, Disseminated Intravascular Coagulation, Hemophagocytic Syndrome,and Hypercalcemia: A Case Report	F	Infant	Not reported	14	Sepsis	No	Discharged	Pulmonary and extra pulmonary Tuberculosis	Microscopic examination of tracheal and gastric aspirates: Positive
Eshiwe et al.	1999	Rare and unusual case of hepatic and disseminated tuberculosis in an immunocompetent patient	F	Adult	Not reported	17	Sepsis	History of tuberculosis	Discharged	Pulmonary and extra pulmonary Tuberculosis	Aspirate of the liver cyst on auramine stains; and PCR and culture: positive for TB
Mohamad et al.	1996	RIPE Treatment Failure in a Patient with *Mycobacterium tuberculosis* sepsis	M	Adult	Not reported	48	Sepsis	No	Discharged	Extrapulmonary tuberculosis	Sputum PCR and culture: Positive
Schroder et al.	2018	Sepsis Syndrome Induced by Tuberculous Perforation of the Esophagus	M	Adult	Not reported	39	Immune thrombocytopenic purpura	No	Death	Pulmonary and extra pulmonary Tuberculosis	Sputum and specimens of pleural empyema microscopy and culture: Positive
Al Argan et al.	2020	Tuberculosis-associated Immune Thrombocytopenia: A Case report	F	Adult	Not reported	46	Aggressive lymphoma	Corticoid or immunomodulation therapies	Discharged	Pulmonary Tuberculosis	Sputum and lymph nodes microscopy and culture: Positive
Reisinger et al.	2020	Tuberculosis sepsis after tocilizumab treatment	M	Adult	Not reported	36	Sepsis	No	Discharged	Extrapulmonary tuberculosis	Ziehle Neelsen staining showed acid-fast rods, and mycobacterial PCR detected high concentrations of *Mycobacterium tuberculosis* DNA complexes in the explanted inguinal lymph node
Sieamann et al.	1998	A Case of Cryptic Miliary Tuberculosis Mimicking Cholecystitis with Sepsis	F	Adult	Not reported	69	Cholecystis	No	Death	Pulmonary and extra pulmonary Tuberculosis	Liver biopsy (post-mortem): identified acid-fast bacilli Blood culture: Positive
Limin et al.	2021	Diagnosis of *Mycobacterium tuberculosis* Septic Shock in Patients with Anti-synthetase Syndrome Based on Next-Generation Sequencing: A Case Report and Literature Review	F	Adult	Not reported	51	Acute suppurative arthritis	Corticoid or immunomodulation therapies	Death	Extrapulmonary tuberculosis	Blood culture: Negative Pleural effusion culture: Negative articular cavity effusion: Negative Blood and articular cavity effusion next-generation sequencing: Positive
Mazade et al.	2001	Congenital tuberculosis presenting as sepsis syndrome: case report and review of the literature	F	Infant	Not reported	34	Sepsis	History of tuberculosis	Discharged	Pulmonary tuberculosis	Blood culture: Negative Tracheal aspirates culture: Positive Tracheal aspirates acid fast bacteria: Positive
Mishra et al.	2019	Tuberculosis septic shock, an elusive pathophysiology and hurdles in management: A case report and review of literature	F	Adult	Not reported	67	Tuberculosis	Hypertension	Death	Pulmonary and extra pulmonary Tuberculosis	Broncho alveolar lavage culture: Positive
Mishra et al.	2019	Tuberculosis septic shock, an elusive pathophysiology and hurdles in management: A case report and review of literature	F	Adult	Not reported	49	Tuberculosis	Hypertension	Death	Pulmonary Tuberculosis	Sputum microscopy, PCR and culture: Positive
Pene et al.	2001	sepsis shocks du to *Mycobacterium tuberculosis* in non-immunocompromised patient	F	Adult	Not reported	69	Sepsis	No	Death	Pulmonary Tuberculosis	Sputum and Ascites culture: Positive
Angoulvant et al.	1998	Septic shock caused by *Mycobacterium tuberculosis* in a non-HIV patient	M	Adult	Not reported	44	Sepsis	No	Death	Extrapulmonary tuberculosis	Bronchoalveolar lavage culture: Positive
Michel et al.	2001	Three cases of septic shock due to tuberculosis without HIV pathology	M	Adult	Not reported	47	Tuberculosis	No	Death	Extrapulmonary tuberculosis	Bronchial aspiration culture: Positive
Michel et al.	2001	Three cases of septic shock due to tuberculosis without HIV pathology	M	Adult	Not reported	41	Pneumothorax	No	Death	Extrapulmonary tuberculosis	Bronchial aspiration culture: Positive
Michel et al.	2001	Three cases of septic shock due to tuberculosis without HIV pathology	F	Adult	Not reported	60	Severe community-acquired pneumonia	Chronic kidney failure	Death	Extrapulmonary tuberculosis	Bronchial aspiration culture: Positive
Colunche et al.	2018	Acute respiratory failure and sepsis due to multidrug-resistant tuberculosis in pregnant midwife	F	Adult	Not reported	20	Sepsis	Pregnancy	Discharged	Pulmonary tuberculosis	Sputum PCR and culture: Positive
Sheldon et al.	2018	Septic shock from disseminated *M. tuberculosis*	M	Adult	Not reported	47	Sepsis	Crohn’s Disease	Discharged	Pulmonary and extra pulmonary Tuberculosis	Sputum culture: Positive Pathology of the terminal ileum: identified acid-fast bacilli
Kathryn et al.	2022	Death of a 29-Year-Old Male from Undifferentiated Sepsis	M	Adult	Not reported	29	Sepsis	No	Death	Pulmonary and extra pulmonary Tuberculosis	Post-mortem pathology of lung, liver, spleen: giant cells, caseous necrosis and PCR confirmation of acid-fast bacilli
Vergara-sanchez et al.	2022	A rare case of disseminated mycobacterial septicaemia (landouzy septicaemia) In an HIV-negative patient	F	Adult	Not reporter	33	Sepsis	No	Death	Pulmonary Tuberculosis	Bronchial aspiration PCR positive
Baljeet et al.	2015	Septic Shock Due to Tuberculosis in Down Syndrome	M	Adult	Yes	16	Sepsis	Down syndrome	Discharged	Pulmonary Tuberculosis	Sputum microscopy examination revealed acid fast bacilli

**Table 2:** Clinical features and underlying conditions in 25 cases of tuberculosis sepsis

Variables	Adult, *N* = 24 (%)	Infant, *N* = 4 (%)
I. History of past illness		
Underlying conditions		
Bone marrow transplantation	1 (4.2)	Not applicable
Chronic kidney failure	1 (4.2)	0 (0)
Cirrhosis	1 (4.2)	Not applicable
Corticoid or immunomodulation therapies	2 (8.3)	0 (0)
Crohn’s disease	1 (4.2)	0 (0)
Down syndrome	1 (4.2)	
History of tuberculosis	1 (4.2)	1 (25)
Hypertension	2 (8.3)	0 (0)
Prematurity	Not applicable	1 (25)
Pregnancy	2 (8.3)	Not applicable
None	12 (50)	2(50)
II. Chief complaint		
Abdominal pain	4 (17)	0 (0)
Confusion	2 (8.3)	0 (0)
Cough	2 (8.3)	0 (0)
Dyspnea	7 (29)	1 (25)
Epistaxis	1 (4.2)	0 (0)
Fever	2 (8.3)	1 (25)
Anorexia	0 (0)	1 (25)
Weakness	6 (25)	1 (25)
III. Symptom and physical examination on admission^a^		
Cough	15 (62)	2 (50)
Night sweats	5 (21)	0 (0)
Diffuse adenopathy	9(43)	2 (50)
Fever	24 (100)	4 (100)
Altered states of consciousness	5 (24)	0 (0)
Hypotension	24(100)	Not reported
Hepatomegaly	5 (21)	2 (50)
Respiratory distress	24 (100)	4 (100)
Weakness	15 (62)	2(50)
IV. Laboratory examinations^*^		
Anemia	9(38)	1 (25)
Leukopenia	12 (50)	3 (75)
Thrombocytopenia	10 (42)	2 (50)
Elevated C-reactive protein	8 (33)	2 (50)

No data was reported for erythrocyte sedimentation rates.

^a^Patients could have more than one sign or abnormality simultaneously; proportions were obtained by dividing the number of patients who showed the sign by the total number of patients (24 for adults and 4 for children)

### Tuberculosis sepsis diagnosis and patient management and treatment outcomes

**Fig. 2:**
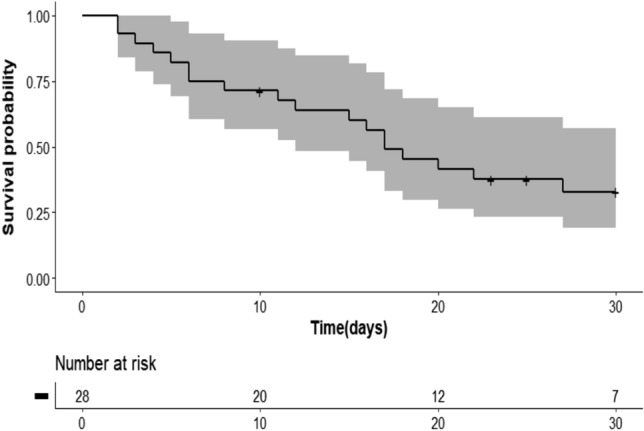
Survival probability patients diagnosed with tuberculosis sepsis

For 75% (3/4) of infants, the initial diagnosis at admission was sepsis, and patients were managed in the intensive care unit; while only 46% (11/24) of adults were diagnosed with sepsis on admission. The empiric anti-tuberculosis was initiated in 53% (15/28) of the patients, and the mean time lapse from presentation to treatment initiation was six days (SD = 9; Table 3). Most (18/28; 64%) patients died within 30 days of the presentation, and in 39% (7/18), the TBS diagnosis was confirmed post-mortem. The diagnosis of tuberculosis was confirmed by either blood culture, extrapulmonary liquid culture, or sputum microscopic, TB molecular diagnosis/culture or pathology of infected organ biopsy (Table 1). The probability of survival of patients diagnosed with tuberculosis sepsis was 68% on day 10; 41% on day 20; and 33% on day 30 after admission, Fig. 2. A higher proportion of death occurred in patients with other diagnoses than sepsis at admission. The mean time (day) of starting empiric anti-tuberculosis treatment since the presentation was 10 (SD = 4) in patients who died, while the treatment was imitated earlier in the patients who survived (mean time = 5, SD = 3).

**Table 3:** Summary of initial diagnosis and length of time between admission and confirmation or suspicion of tuberculosis sepsis diagnosis

Variables	Adults, *N* = 24(%)	Infants, *N* = 4(%)
Initial diagnosis		
Acute suppurative arthritis	1 (4.2)	0 (0)
Aggressive lymphoma	1 (4.2)	0 (0)
Cholecystitis	1 (4.2)	0 (0)
Chorioamnionitis	1 (4.2)	0 (0)
Cytomegalovirus reactivation	1 (4.2)	0 (0)
Immune thrombocytopenic purpura	1 (4.2)	0 (0)
Peritonitis	1 (4.2)	1 (25)
Pneumothorax	1 (4.2)	0 (0)
Sepsis	11 (46)	3 (75)
Severe community-acquired pneumonia	1 (4.2)	0 (0)
Tuberculosis	4 (17)	0 (0)
Time of TB sepsis diagnosis or suspicion (number of days after admission when the sepsis diagnosis was done), (SD)	6 (9)	4 (2)
Treatment outcome		
Death	17 (71)	1 (25)
Discharged	7 (29)	3 (75)

*SD* standard deviation

## Discussion

We performed a systematic review and meta-analysis of published cases of tuberculosis sepsis in HIV-negative patients from 1991, up to September 25, 2022. The major findings were that the half of patients did not report known underline immunocompromised co-morbidity, 61% (17/28) reported cough at admission, 64% (18/28) died within 30 days since presentation and the TBS diagnosis was confirmed only at post-mortem for 39% (7/18) of the patients who died. A higher proportion of death occurred in adult patients with delayed initiation of anti-tuberculosis treatment.

The major challenge of tuberculosis sepsis is the delay of diagnosis or difficulty of its recognition by clinicians. Our study confirms that there are no specific signs or symptoms of TBS and that patients present with the common sign of sepsis. Recent research and advocacy improve the awareness of clinicians on tuberculosis bacteraemia and/or sepsis in HIV patients [44–47]. However, our review shows that tuberculosis sepsis can occur in patients without HIV infection or known co-morbidities. Therefore, TBS should be investigated in any tuberculosis patient presenting sepsis signs irrespective of HIV status. In highly TB- endemic settings, we recommend broadening the investigation of the etiology of sepsis to *Mycobacterium tuberculosis.* The culture of samples is currently the gold standard for tuberculosis diagnosis. However, culture takes two to six weeks to be reported. Therefore, rapid molecular diagnostic such as GeneXpert is suggested in the purpose to allow as soon as possible anti-tuberculosis treatment. However, the pulmonary manifestations were not common in all cases of TBS and *the M. tuberculosis* is not identifiable all-time in the sputum. The sensibility of GeneXpert in extrapulmonary samples ranged from 55.2 to 69.9% [48–50]. Barr et al. suggested that a combination of sputum GeneXpert, blood culture and urine lipoarabinomannan, could improve the diagnostic yield of TB in critically ill adult patients [14]. There is a need to improve the diagnostic tools for disseminated tuberculosis which increase the risk to develop TBS. A risk score derived from a model containing independent predictors has been suggested; however, it was derived from patients with HIV infection only and needs to be validated in other settings [7]. The clinical feature of TBS does not seem to be different according to the HIV infection status of the patients. Therefore, the clinician should keep in mind the alternative diagnosis of TBS in HIV-negative patients with tuberculosis signs associated with organ failure manifestations. With BCG vaccination protecting from TB meningitis and sepsis at least at a younger age, it would be important if future reports would include this information routinely.

### Strengths and weaknesses

Using a systematic search strategy in four widely used databases, we increased the chance to identify all case reports of tuberculosis sepsis. Despite having applied refined selection inclusion criteria, there is the possibility of missing some important case reports. Publication bias is another weakness of case report review since only rare and untypical observations are more likely to be published. The publication of case reports also depends on the research experience and ability of the clinician in charge of the case seeming to be unusual. The BCG vaccination status was reported only in one case report. The proportion of TBS cases without known co-morbidity is impressive. Since this is a summary of case reports published, we cannot ensure that an in-depth investigation (including further laboratory assessments) will confirm or refute the absence of co-morbidities in these patients. Therefore, the interpretation of our findings should be done with caution. The role of radiology imaging in the diagnosis of not bacteriological confirmed is well known. We did not extract such information from the articles included. Despite these limitations, our review is of clinical practice and research implications interest because findings from this review contribute to improving the awareness of clinicians on the clinical feature of tuberculosis sepsis and showed that it could occur in infant and adult patients irrespective of HIV infection status.

## Conclusions

TBS is reported as a case report because of its rare incidence in many settings. It is not usually suspected in the first place, inducing a risk of delayed diagnosis. Most case reports had death as an outcome and the mortality rate is more frequent in groups of patients where the diagnosis of TBS was not suspected at admission. In an endemic setting, TBS should be envisaged in patients with tuberculosis likely symptoms presenting sepsis signs as well. Empiric anti-tuberculosis treatment should be initiated as soon as possible.

## Supplementary Information

Below is the link to the electronic supplementary material.

Supplementary file1 (DOCX 43 KB)

## Data Availability

All relevant data have been published in the manuscript or in the supplementary material. Further details can be obtained by writing to the corresponding author.
