# Supplementary material for: Clinical features, treatment outcomes and mortality risk of tuberculosis sepsis in HIV-negative patients: a systematic review and meta-analysis of case reports
Source: Infection. 2022 Nov 16;51(3):609–21. doi: 10.1007/s15010-022-01950-4 (PMC10205828; doi:10.1007/s15010-022-01950-4)
Supplement: Supplementary file 1 — Supplementary file1 (DOCX 43 KB) [file 15010_2022_1950_MOESM1_ESM.docx]

**Supplementary Material**

**Supplementary File S1: Search strategy**

**Supplementary File S 2: Overview of excluded studies**

**Supplementary File S3: Risk of bias assessment**

Supplementary File S1: Search strategy

## PubMed

("tuberculosis"[All Fields] OR "tuberculosis"[MeSH Terms] OR "tuberculosis"[All Fields] OR "tuberculoses"[All Fields] OR "tuberculosis s"[All Fields]) AND ("sepsis"[MeSH Terms] OR "sepsis"[All Fields]) AND ((("ieee int conf automation sci eng case"[Journal] OR "case phila"[Journal] OR "case"[All Fields]) AND "report*"[All Fields]) OR (("ieee int conf automation sci eng case"[Journal] OR "case phila"[Journal] OR "case"[All Fields]) AND "serie*"[All Fields]))

## Google Scholar

(tuberculosis or Mycobacterium) AND sepsis AND (case series report OR case)

## Web of Science (core collection)

**Tuberculosis sepsis** (All Fields) and **case report OR case series** (All Fields)

## Embase

('tuberculosis sepsis' OR (('tuberculosis'/exp OR tuberculosis) AND ('sepsis'/exp OR sepsis))) AND ('case report'/exp OR 'case report' OR (case AND report) OR 'case series' OR (case AND series))

Supplementary File S2: Overview of excluded studies

| Authors | Years | Title | Reason for exclusion |
| --- | --- | --- | --- |
| Toyoda et al | 2010 | A case of severe pulmonary tuberculosis with septic shock and ARDS | Not in English or French |
| Kanabus et al | 1970 | A case of tuberculosis diagnosed in the course of staphylococcal septicaemia in premature infants | Not tuberculosis sepsis |
| Karinauske et al | 2018 | A case report and literature review: previously excluded tuberculosis masked by amiodarone-induced lung injury | Not tuberculosis sepsis |
| T. R. Nunnet al | 2022 | A prospective study of pyogenic sepsis of the hip in childhood | Not a case report |
| Sehgal et al | 2021 | A Randomized Trial of Mycobacterium w in Severe Presumed Gram-Negative Sepsis | Not a case report |
| Gaubert et al | 1958 | A rare form of primary infection; tuberculous septicemia | Not tuberculosis sepsis |
| Kowalewski et al | 2017 | Abdominal tuberculosis after removal of an adjustable gastric band - report of an unusual case | Not tuberculosis sepsis |
| Islam et al | 2017 | Abdominal tuberculosis and spontaneous miscarriage | Not tuberculosis sepsis |
| Fu et al | 2020 | Abdominal Tuberculosis Managed Surgically in the Late Phase: A Case Report | Not tuberculosis sepsis |
| Frame et al | 1987 | Active tuberculosis in the medical intensive care unit: a 15-year retrospective analysis | Not a case report |
| Arum et al | 2022 | Acute Perforation of Small Intestine Due to Tuberculosis - Kakar - 1983 - Australia and New Zealand | Not tuberculosis sepsis |
| Lee et al | 2011 | Acute respiratory distress syndrome caused by miliary tuberculosis: a multicentre survey in South Korea | Not a case report |
| Peng et al | 2022 | Analysis of 170 cases of congenital TB reported in the literature between 1946 and 2009 - | Not a case report |
| Barber et al | 1990 | Bacteraemia due to Mycobacterium tuberculosis in patients with human immunodeficiency virus infection: A report of 9 cases and a review of the literature | Not in English or French |
| Joseph et al | 2012 | Bcg sepsis following intravesical bcg administration for the treatment of bladder cancer | Following BCG injection |
| Widger et al | 2010 | Breast milk causing neonatal sepsis and death | Not tuberculosis sepsis |
| Spronk et al | 2019 | Calculating incidence rates and prevalence proportions: not as simple as it seems | Not a case report |
| Kosheva et al | 1985 | Case of acute leukaemia complicated by tuberculous sepsis | Not in English or French |
| Rosas et al | 2007 | CD14 C(-159)T Polymorphism Is a Risk Factor for Development of Pulmonary Tuberculosis | Not a case report |
| Hogg et al | 1999 | Central Line Sepsis in a Child Due to a Previously Unidentified Mycobacterium | Not tuberculosis sepsis |
| Erbes et al | 2022 | Characteristics and outcome of patients with active pulmonary tuberculosis requiring intensive care | Not a case report |
| Hong et al | 2003 | Characterization of a Novel Rapidly Growing Mycobacterium Species Associated with Sepsis | Not a case report |
| Herné et al | 1980 | Chronic lymphocytic leukaemia, acute anergic tuberculosis, multiple caseous adenitis | Not tuberculosis sepsis |
| Byashalira et al | 2022 | Clinical outcomes of new algorithm for diagnosis and treatment of Tuberculosis sepsis in HIV patients | Not a case report |
| Aguado et al | 1997 | Clinical Presentation and Outcome of Tuberculosis in Kidney, Liver, And Heart Transplant Recipients in Spain1 | Not a case report |
| Dudaka et al | 2020 | Coinfection of Typhoid Fever with Tuberculosis: A Challenge to Surgical Management | Not a case report |
| Sherwood et al | 2005 | Completion pneumonectomy for chronic mycobacterial disease | Not a case report |
| Oliveira de Araujo et al | 2021 | Complicated Lumbar Tuberculous Spondylodiscitis In Disseminated Tuberculosis, Treated Using A Non-Conventional Anterior Support System For Hydrostatic Distraction: A Case Report | Not tuberculosis sepsis |
| Shah et al | 2014 | Complications of tuberculosis | Not a case report |
| Kini et al | 2002 | Congenital tuberculosis associated with maternal asymptomatic endometrial tuberculosis | Not tuberculosis sepsis |
| Sosa et al | 2007 | Congenital tuberculosis associated with maternal disseminated miliary tuberculosis | Not in English or French |
| Ira et al | 2022 | Consumption Coagulopathy in Miliary Tuberculosis Annals of Internal Medicine | Not tuberculosis sepsis |
| Baldini et al | 1988 | Deep sepsis from mycobacterium tuberculosis after total hip replacement | Not tuberculosis sepsis |
| Xie et al | 2022 | Differential Adverse Event Profiles Associated with BCG as a Preventive Tuberculosis Vaccine or Therapeutic Bladder Cancer Vaccine Identified by Comparative Ontology-Based VAERS and Literature Meta-Analysis | Not a case report |
| Ziegler et al | 2018 | Disseminated Mycobacterium bovis infection post-kidney transplant following remote intravesical BCG therapy for bladder cancer | Following BCG injection |
| Kerkhoff et al | 2017 | Disseminated tuberculosis among hospitalised HIV patients in South Africa: a common condition that can be rapidly diagnosed using urine-based assays | Not a case report |
| Kandemir et al | 2003 | Elevation of procalcitonin level in patients with pulmonary tuberculosis and in medical staff with close patient contact | Not a case report |
| Jones et al | 2010 | Aetiology of Illness in Patients with Severe Sepsis Admitted to the Hospital from the Emergency Department | Not tuberculosis sepsis |
| Ritesh et al | 2022 | Experience with ARDS caused by tuberculosis in a respiratory intensive care unit | Not a case report |
| Hensel et al | 2013 | Fatal outcome of multiorgan tuberculosis with peritoneal involvement after abdominal surgery | Not in English or French |
| Bofinger et al | 2007 | Fever of Unknown Origin Caused by Tuberculosis | Not a tuberculosis sepsis |
| Oladiran et al | 2022 | Full article: Disseminated BCG sepsis following intravesical therapy for Bladder Carcinoma: A case report and review of literature | Following BCG injection |
| Padhi et al | 2013 | Hemophagocytic lymph histiocytosis: critical reappraisal of a potentially under-recognized condition | Not tuberculosis sepsis |
| Em et al | 2012 | Hepatic tuberculosis presenting with extreme hyperserotonaemia masquerading as adult-onset Still's disease: a case report | Not tuberculosis sepsis |
| Koh et al | 2013 | Host Responses to Melioidosis and Tuberculosis Are Both Dominated by Interferon-Mediated Signalling | Not tuberculosis sepsis |
| Vandenbroucke et al | 2012 | Incidence rates in dynamic populations | Not a case report |
| Dettmeyer et al | 2018 | Lethal Infections, Sepsis, and Shock | Not a case report |
| Gardner et al | 1949 | Lymphocytic Leukemoid Reaction Of The Blood Associated With Miliary Tuberculosis | Not tuberculosis sepsis |
| van et al | 2010 | Maternal sepsis: epidemiology, aetiology and outcome | Not a case report |
| Colmenero et al | 2012 | Miliary pulmonary tuberculosis following intravesical BCG therapy: case report and literature review | Following BCG injection |
| Stanojevic et al | 2018 | Miliary tuberculosis complicated by staphylococcal sepsis | Not tuberculosis sepsis |
| Jog et al | 2011 | Mycobacterial Sepsis and Multiorgan Failure Syndrome | Not a case report |
| Legout et al | 2001 | Mycobacterial sepsis following instillation of intravesical bacillus. Is corticosteroid therapy necessary? | Following BCG injection |
| Thamthitiwat et al | 2011 | Mycobacterium bovis bacteremia in immunocompetent neonates following vaccination | Following BCG injection |
| Jacob et al | 2013 | Mycobacterium tuberculosis Bacteraemia in a Cohort of HIV-Infected Patients Hospitalized with Severe Sepsis | Not a case report |
| Kethireddy et al | 2013 | Mycobacterium tuberculosis Septic Shock | Not a case report |
| Figueroa et al | 2001 | Neonatal Outcome of Children Born to Women with Tuberculosis | Not a tuberculosis sepsis |
| Mehta et al | 2004 | Ocular lesions in acute disseminated tuberculosis | Not a tuberculosis sepsis |
| SÃ¡enz et al | 2015 | Perinatal tuberculosis | Not in English or French |
| Jin et al | 2010 | Procalcitonin: Uses in the Clinical Laboratory for the Diagnosis of Sepsis | Not a case report |
| Kim et al | 2008 | Pulmonary tuberculosis with acute respiratory failure | Not a case report |
| Trauner et al | 1995 | Recurrent Salmonella enteritidis sepsis and hepatic tuberculosis. | Not tuberculosis sepsis |
| Cf et al | 2020 | Rifampicin induced shock during re-exposure for treatment of latent tuberculosis | Not tuberculosis sepsis |
| Giamarellos et al | 2012 | Risk assessment in sepsis: a new prognostication rule by APACHE II score and serum soluble urokinase plasminogen activator receptor | Not tuberculosis sepsis |
| Elton et al | 2015 | Sepsis in obstetrics | Not a case report |
| Gardner et al | 2009 | Sepsis in the Neonate | Not a case report |
| Japiass et al | 2010 | Sepsis is a major determinant of outcome in critically ill HIV/AIDS patients | Not a case report |
| Rafael Silva et al | 2011 | Sepsis tuberculosa gravissima | Not in English or French |
| Fichte et al | 2018 | Septic shock in a female patient with miliary tuberculosis | Not in English or French |
| Gachot et al | 1990 | Severe tuberculosis in patients with human immunodeficiency virus infection | Not tuberculosis sepsis |
| Dacombe et al | 2013 | Stage 3 pyomyositis of the gluteus minimums; Staphylococcus aureus sepsis, auto anticoagulation, proximal femoral osteomyelitis and the role of surgical intervention | Not tuberculosis sepsis |
| Morales et al | 2007 | Successful Recovery After Disseminated Infection Due to Mycobacterium Abscesses in a Lung Transplant Patient: Subcutaneous Nodule as First Manifestation | Not tuberculosis sepsis |
| Dewan et al | 2010 | Surgery for pulmonary tuberculosis | Not tuberculosis sepsis |
| Ye et al | 2019 | The clinical characteristics of patients with sepsis in a tertiary referral hospital in Yangon, Myanmar | Not tuberculosis sepsis |
| Kestler et al | 2013 | The development of an emergency sepsis care algorithme in Botswana | Not tuberculosis sepsis |
| Pillay et al | 2001 | The increasing burden of tuberculosis in pregnant women, new-borns and infants under 6 months of age in Durban, KwaZulu Natal | Not tuberculosis sepsis |
| Tanaka et al | 2019 | The most common causative bacteria in maternal sepsis-related deaths in Japan were group A Streptococcus: A nationwide survey | Not tuberculosis sepsis |
| Seymour et al | 2017 | Time to Treatment and Mortality during Mandated Emergency Care for Sepsis | Not tuberculosis sepsis |
| Alkhuja et al | 2001 | Tuberculosis and sudden death: A case report and review | Not tuberculosis sepsis |
| Lanoix et al | 2014 | Tuberculosis in the intensive care unit: a descriptive analysis in a low-burden country | Not a case report |
| Babhulkar et al | 2022 | Tuberculosis of the Hip: Clinical Orthopaedics and Related Research | Not tuberculosis sepsis |
| Castro et al | 2007 | Tuberculosis Surveillance: Data for Decision-Making | Not tuberculosis sepsis |
| Ündar et al | 2006 | Tuberculosis-Associated Hemophagocytic Syndrome: A Report of Two Cases and a Review of the Literature | Not tuberculosis sepsis |
| Hauch et al | 2020 | Tuberculosis-Associated HLH in an 8-Month-Old Infant: A Case Report and Review | Not tuberculosis sepsis |
| Ekaterina et al | 2011 | Urogenital Tuberculosis: Classification, Diagnosis, and Treatment - ScienceDirect | Not tuberculosis sepsis |
| Zhe Zhe et al | 2022 | Mycobacterium tuberculosis bacteraemia in a human immunodeficiency virus-negative patient with liver cirrhosis: A case report | Not tuberculosis sepsis |
| Ahmed et all | 2022 | Acute Cholecystitis Presenting With Septic Shock | Not tuberculosis sepsis |
|  |  | as the First Presentation in an Elderly Patient |  |
| Pía Iglesias | 2021 | Acute cholecystitis, septic shock, and miliary tuberculosis | Not in English or French |
| Audulev et al | 1984 | Acute tuberculous sepsis | Not in English or French |
| Pesce et al | 1999 | Acute tuberculous septicaemia. | Not a tuberculosis sepsis |
| Kassapidis | 2020 | Diagnosing mycobacterium tuberculosis bacteraemia in an immunocompromised female | HIV positive patient |
| Vadilo et al | 1994 | AIDS presenting as septic shock caused by mycobacterium tuberculosis | HIV positive patient |
| Grigoru et al | 2008 | Disseminated tuberculosis with severe multi- organ failure in a patient with Aids | HIV positive patient |
| Nyirjesy et al | 1993 | Fulminant tuberculosis complicating pregnancy in a patient infected with the human immunodeficiency virus | HIV positive patient |
| Rodriguez | 1997 | Septic shock and multiple organ failure caused by Mycobacterium tuberculosis | Not in English or French |
| Cordtz | 2005 | Severe and sudden progress of septic shock related to infection with M. tuberculosis | Not in English or French |
| Silva et al | 2011 | Severe disseminated tuberculosis in a patient on immunosuppressive treatment. Report of one case | Not in English or French |
| Phelippeau et al | 2015 | Severe pulmonary tuberculosis in the ICU, diagnosis and treatment | Not a case report |
| Dziwiński et al | 1970 | Severe tuberculous septicemia | Not in English or French |
| Barmes et al | 1987 | Six Cases of Mycobacterium tuberculosis Bacteremia | Not a tuberculosis sepsis |
| X. Xiao | 2020 | Tuberculosis in patients with systemic lupus erythematosus–a 37-year longitudinal survey-based study | Not a case report |
| Mueller et all | 1980 | Unrecognized atypical tuberculosepsis in generalized hematologic neoplasms | Not in English or French |
| Khosa et al | 2022 | Tuberculous Tamponade With A Twist: A Case Of Tb And Covid-19 | Not tuberculosis sepsis |
| Takeshi et al | 2022 | Uncommon Presentation of Tuberculosis as an Incidentally Discovered Solitary Pleural Tuberculoma | Not a tuberculosis sepsis |
| Tschöp et al | 2012 | Tuberculous encephalitis, Landouzy sepsis and Pott's disease. Complications after surgery for spinal stenosis | Not in English or French |
| Pasculle et al | 1991 | Tuberculous bacillemia, hyperpyrexia, and rapid death | Not tuberculosis sepsis |
| Naumov et al | 1997 | Tuberculous sepsis in expert practice | Not a case report |
| Krishnasamy et al | 2013 | Tuberculous pyomyositis: A rare but serious diagnosis | Not tuberculosis sepsis |
| Campo et al | 1996 | tuberculosis-associated hemophagocytic syndrome: A systemic process | Not a tuberculosis sepsis |
| Humbert et al | 2015 | Sepsis or not sepsis? The difficult diagnosis of azathioprine hypersensibility. A case report | Not a tuberculosis sepsis |
| Costanzo | 2022 | A rare presentation of tb-related septic shock | Abstract /conference publication with lack of information on inclusion criteria |
| Fiyad haniff | 2022 | A shocking case of disseminated tb | Abstract /conference publication with lack of information on inclusion criteria |
| Lauren Old et al | 2020 | a very rare presentation of miliary tuberculosis in mid-trimester pregnancy masquerading as sepsis and severe acute respiratory syndrome category: clinical lesson | Abstract /conference publication with lack of information on inclusion criteria |
| Debbie et al | 2006 | Severe Tuberculosis Sepsis in an Immunocompetent Patient | Abstract /conference publication with lack of information on inclusion criteria |
| Stephanie Hametner et al | 2013 | Tuberculous sepsis during antiviral HCV triple therapy | Abstract /conference publication with lack of information on inclusion criteria |
| Myles Rowe et al | 2015 | An unusual case of sepsis? A rare presentation of a common disease | Abstract /conference publication with lack of information on inclusion criteria |

**Supplementary File S3**: Risk of bias assessment

| **Study** |  |  |  |  | Criteria |  |  |  |  |
| --- | --- | --- | --- | --- | --- | --- | --- | --- | --- |
|  | 1. Were patient’s demographic characteristics clearly described? | 2. Was the patient’s history clearly described and presented as a timeline? | 3. Was the current clinical condition of the patient on presentation clearly described? | 4. Were diagnostic tests or assessment methods and the results clearly described? | 5. Was the intervention(s) or treatment procedure(s) clearly described? | 6. Was the post-intervention clinical condition clearly described? | 7. Were adverse events (harms) or unanticipated events identified and described? | 8. Does the case report provide takeaway lessons? | Overall appraisal |
| Artsiom et al | Yes | Yes | Yes | Yes | Yes | No | Not applicable | Yes | Include |
| Nakbanpot et al | Yes | No | Yes | Yes | Yes | Yes | Not applicable | Yes | Include |
| Barbosa et al | Yes | Yes | Yes | Yes | Yes | Yes | Not applicable | Yes | Include |
| Chun-Yuan et al | Yes | No | Yes | Yes | Yes | Yes | Not applicable | Yes | Include |
| Kindler et al | Yes | Yes | Yes | Yes | Yes | No | Not applicable | Yes | Include |
| Mitchon et al | Yes | Yes | Yes | Yes | Yes | Yes | Not applicable | Yes | Include |
| Sydow et al | Yes | Yes | Yes | Yes | Yes | Yes | Not applicable | Yes | Include |
| Okascharoenet al | Yes | Yes | Yes | Yes | Yes | Yes | Not applicable | Yes | Include |
| Eshiwe et al | Yes | Yes | Yes | Yes | Yes | Yes | Not applicable | Yes | Include |
| Mohamad et al | Yes | Yes | Yes | Yes | Yes | Yes | Not applicable | Yes | Include |
| Schroder et al | Yes | Yes | Yes | Yes | Yes | Yes | Not applicable | Yes | Include |
| Al Argan et al | Yes | Yes | Yes | Yes | Yes | Yes | Not applicable | Yes | Include |
| Reisinger et al | Yes | Yes | Yes | Yes | Yes | Yes | Not applicable | Yes | Include |
| sieamann et al | Yes | Yes | Yes | Yes | Yes | Yes | Not applicable | Yes | Include |
| Limin et al | Yes | Yes | Yes | Yes | Yes | Yes | Not applicable | Yes | Include |
| Mazade et al | Yes | Yes | Yes | Yes | Yes | Yes | Not applicable | Yes | Include |
| Mishra et al | Yes | Yes | Yes | Yes | Yes | Yes | Not applicable | Yes | Include |
| Mishra et al | Yes | Yes | Yes | Yes | Yes | Yes | Not applicable | Yes | Include |
| Pene et al | Yes | Yes | Yes | Yes | Yes | Yes | Not applicable | Yes | Include |
| Angoulvant et al | Yes | Yes | Yes | Yes | Yes | Yes | Not applicable | Yes | Include |
| Michel et al | Yes | Yes | Yes | Yes | Yes | Yes | Not applicable | Yes | Include |
| Michel et al | Yes | Yes | Yes | Yes | Yes | Yes | Not applicable | Yes | Include |
| Michel et al | Yes | Yes | Yes | Yes | Yes | Yes | Not applicable | Yes | Include |
| Colunche et al | Yes | Yes | Yes | Yes | Yes | No | Not applicable | Yes | Include |
| Sheldon et al | Yes | Yes | Yes | Yes | Yes | No | Not applicable | Yes | Include |
| Kathryn et al | Yes | Yes | Yes | Yes | Yes | No | Not applicable | No | Include |
| Vergara-Sanchez et al | Yes | Yes | Yes | Yes | Yes | No | Not applicable | No | Include |
| Baljeet et al | Yes | Yes | Yes | Yes | Yes | No | Not applicable | No | Include |
